# Potential Survival Benefit of Anti-Apoptosis Protein: Survivin-Derived Peptide Vaccine with and without Interferon Alpha Therapy for Patients with Advanced or Recurrent Urothelial Cancer—Results from Phase I Clinical Trials

**DOI:** 10.1155/2013/262967

**Published:** 2013-11-20

**Authors:** Toshiaki Tanaka, Hiroshi Kitamura, Ryuta Inoue, Sachiyo Nishida, Akari Takahashi-Takaya, Sachiyo Kawami, Toshihiko Torigoe, Yoshihiko Hirohashi, Taiji Tsukamoto, Noriyuki Sato, Naoya Masumori

**Affiliations:** ^1^Department of Urology, Sapporo Medical University School of Medicine, South-1, West-16, Chuo-ku, Sapporo 060-8543, Japan; ^2^1st Department of Pathology, Sapporo Medical University School of Medicine, South-1, West-17, Chuo-ku, Sapporo 060-8556, Japan

## Abstract

We previously identified a human leukocyte antigen (HLA)-A24-restricted antigenic peptide, survivin-2B80–88, a member of the inhibitor of apoptosis protein family, recognized by CD8+cytotoxic T lymphocytes (CTL). In a phase I clinical trial of survivin-2B80-88 vaccination for metastatic urothelial cancer (MUC), we achieved clinical and immunological responses with safety. Moreover, our previous study indicated that interferon alpha (IFN**α**) enhanced the effects of the vaccine for colorectal cancer. Therefore, we started a new phase I clinical trial of survivin-2B80–88 vaccination with IFN**α** for MUC patients. Twenty-one patients were enrolled and no severe adverse event was observed. HLA-A24/survivin-2B80–88 tetramer analysis and ELISPOT assay revealed a significant increase in the frequency of the peptide-specific CTLs after vaccination in nine patients. Six patients had stable disease. The effects of IFN**α** on the vaccination were unclear for MUC. Throughout two trials, 30 MUO patients received survivin-2B80–88 vaccination. Patients receiving the vaccination had significantly better overall survival than a comparable control group of MUO patients without vaccination (*P* = 0.0009). Survivin-2B80–88 vaccination may be a promising therapy for selected patients with MUC refractory to standard chemotherapy. This trial was registered with UMIN00005859.

## 1. Introduction

Urothelial carcinoma of the bladder is the fourth most common cancer in men [1]. Systemic chemotherapy has been the mainstay of management for metastatic urothelial cancer [2, 3], and cisplatin-based combinations have evolved as the standard first-line therapy. The regimens consisting of methotrexate, vinblastine, doxorubicin, and cisplatin (MVAC) and gemcitabine and cisplatin (GC) are currently employed and provide prolongation of survival up to 14.8 and 13.8 months, respectively [3]. However, no standard therapy has been established for patients with progressive disease after the first-line chemotherapy [2, 3], and some new regimens including other anticancerous agents such as paclitaxel, ifosphamide, nedaplatin, and vinflunine are used in this setting [4–6], although they have not been proven to have sufficient clinical efficacy.

On the other hand, during the past two decades, research on human tumor immunology and cancer immunotherapy has progressed. Immunization with peptides derived from cancer-specific antigen induces antitumor cytotoxic T lymphocytes (CTLs) [7–9]. A large number of cancer-specific antigens have been identified from melanomas and other cancers, and clinical trials of peptide-based immunotherapy have been carried out.

We previously reported that survivin and its splicing variant survivin-2B were expressed abundantly in various cancer tissues and cancer cell lines, including urothelial cancer, and were suitable as target antigens for active-specific anticancer immunization [10]. Subsequently, we identified the human leukocyte antigen (HLA)-A24-restricted antigenic peptide survivin-2B80–88 (AYACNTSTL) derived from the exon 2B-encoded region and recognized by CTLs in the context of HLA-A24 molecules. In addition, we reported further evidence that the survivin-2B80–88 peptide might serve as a potent immunogenic cancer vaccine for various cancers, including bladder cancer [11]. On the basis of these studies, we started a phase I clinical study using survivin-2B80–88 peptide vaccination for urothelial cancers (Study 1) [12]. This study revealed that survivin-2B80–88 peptide vaccination was safe and well tolerated without severe side effects and could induce survivin-2B80–88 peptide-specific CTLs. Moreover, we previously reported that combination with interferon (IFN) alpha successfully enhanced the immunological responses of patients who received survivin-2B80–88 peptide vaccination for colorectal [13] and pancreatic cancers [14]. Therefore we conducted a phase I clinical study of survivin-2B80–88 peptide vaccination in combination with IFN alpha for patients with advanced or recurrent urothelial cancer expressing survivin to assess the safety and immunological efficacy (Study 2). In addition, we analyzed the effects on survival of survivin-2B80–88 peptide vaccination therapy with and without IFN alpha using the pooled data of Study 1 and Study 2.

## 2. Materials and Methods

### 2.1. Patient Selection

The study protocol was approved by the Clinical Institutional Ethical Review Board of the Medical Institute of Bioregulation, Sapporo Medical University, Japan. The HLA-A typing and immunohistochemical study were performed after obtaining informed consent from all candidate patients. Patients enrolled in this study were required to conform to the following criteria: (1) histologically proven urothelial cancer, (2) HLA-A∗2402 positive, (3) survivin- and HLA class I-positive carcinomatous lesions on the primary site demonstrated by immunohistochemistry, (4) age between 20 and 85 years old, (5) surgical excision of the primary tumor, and (6) Eastern Cooperative Oncology Group (ECOG) performance status between 0 and 3. Exclusion criteria included (1) prior cancer therapy such as chemotherapy, radiation therapy, steroid therapy, or other immunotherapies within the previous 4 weeks, (2) the presence of other cancers that might influence the prognosis, (3) immunodeficiency or a history of splenectomy, (4) severe cardiac insufficiency, acute infection, or hematopoietic failure, and (5) unsuitability for the trial based on clinical judgment. This study was carried out at the Department of Urology, Sapporo Medical University Hospital from May 2009 to June 2013.

### 2.2. Peptide Preparation

The peptide, survivin-2B80–88 with the sequence AYACNTSTL, was prepared under good manufacturing practice conditions by Multiple Peptide Systems (San Diego, CA, USA) [12–14]. The identity of the peptide was confirmed by mass spectrometry analysis and the purity was shown to be more than 98% as assessed by high-pressure liquid chromatography analysis. The peptide was supplied as a freeze-dried, sterile white powder. It was dissolved in 1.0 mL of physiological saline (Otsuka Pharmaceutical Co., Ltd, Tokyo, Japan) and stored at −80°C until just before use.

### 2.3. IFA and IFN Alpha Preparation

Montanide ISA 51 (Seppic, Paris, France) was used as IFA. Human IFN alpha was purchased from Dainippon-Sumitomo Pharmaceutical Co. (Osaka, Japan).

### 2.4. Patient Treatment

**Figure 1:**
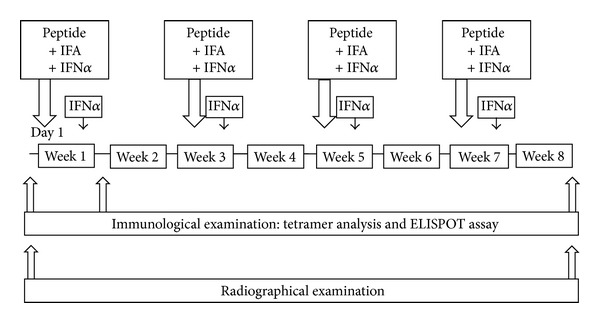
Protocol of Study 2. The protocol consisted of survivin-2B80–88 peptide, IFA, and IFN*α*. IFA: incomplete Freund's adjuvant; IFN: interferon.

In Study 1 we administered the survivin-2B80–88 peptide plus IFA [12]. In Study 2, the survivin-2B80–88 peptide plus IFA and a type-I IFN, IFN alpha, were used as illustrated in Figure 1. The doses were determined according to previous studies [13, 14]. Survivin-2B80–88 at a dose of 1 mg/1 mL and IFA at a dose of 1 mL were mixed immediately before vaccination. The patients were then vaccinated subcutaneously four times at 14-day intervals. In addition, IFN alpha at a dose of 3,000,000 IU was administered subcutaneously immediately before vaccination and three days after vaccination at the site of vaccination. The primary endpoint was safety. The secondary endpoints were investigations about antitumor effects and clinical and immunological monitoring.

### 2.5. Toxicity Evaluation

Patients were examined closely for signs of toxicity during and after vaccination. Adverse events were recorded using the National Cancer Institute Common Terminology Criteria for Adverse Events version 4.0 (CTCAE v4.0) [15].

### 2.6. Clinical Response Evaluation

Physical examinations and hematological examinations were conducted before and after each vaccination [12–14]. Immunohistochemical study of the HLA class I expression in patients' primary urothelial cancer tissues was done with anti-HLA class I heavy chain monoclonal antibody EMR-8-5 (Funakoshi Co., Tokyo, Japan). We evaluated tumor size using CT scans or MRI by comparing the size before the first vaccination with that after the fourth vaccination. A complete response (CR) was defined as complete disappearance of all measurable and evaluable diseases. A partial response (PR) was defined as a ≥30% decrease from baseline in the size of all measurable lesions (sum of maximal diameters). Progressive disease (PD) was defined as an increase in the sum of maximal diameters by at least 20% or the appearance of new lesions. Stable disease (SD) was defined as the absence of criteria matching those for CR, PR, or PD [12–14].

### 2.7. *In Vitro* Stimulation of PBMC

PBMCs were isolated from blood samples by Ficoll-Conray density gradient centrifugation. They were then frozen and stored at −80°C. As needed, frozen PBMCs were thawed and incubated in the presence of 30 *μ*g/mL survivin-2B80–88 in AIM-V medium containing 10% human serum at room temperature. Next, interleukin-2 was added at a final concentration of 50 U/mL 1 h, 2 days, 4 days, and 6 days after the addition of the peptide. On day 7 of culture, the PBMCs were analyzed by tetramer staining and ELISPOT assay.

### 2.8. Tetramer Staining

FITC-labeled HLA-A∗2402-human immunodeficiency virus (HIV) peptide (RYLRDQQLL) and PE-labeled HLA-A∗2402-survivin-2B80–88 peptide tetramers were purchased from MBL, Inc. (Nagoya, Japan). For flow cytometric analysis, PBMCs, which were stimulated in vitro as above, were stained with the PE-labeled tetramer at 37°C for 20 min, followed by staining with an FITC-conjugated anti-CD8 mAb (Beckton Dickinson Biosciences, San Jose, CA, USA) at 4°C for 30 min. Cells were washed twice with PBS before fixation in 1% formaldehyde. Flow cytometric analysis was performed using FACSCalibur and CellQuest software (Beckton Dickinson Biosciences, San Jose, CA, USA). The frequency of CTL precursors was calculated as the number of tetramer-positive cells divided by the number of CD8-positive cells [12–14].

### 2.9. ELISPOT Assay

ELISPOT plates were coated sterilely overnight with an IFN-c capture antibody (Beckton Dickinson Biosciences) at 4°C. The plates were then washed once and blocked with AIM-V medium containing 10% human serum for 2 h at room temperature. CD8-positive T cells separated from patients' PBMC (5 × 10^3^ cells/well), which were stimulated in vitro as above, were then added to each well along with HLA-A24-transfected CIR cells (CIR-A24) (5 × 10^4^ cells/well), which had been preincubated with or without survivin-2B80–88 (10 mg/mL) or with an HIV peptide as a negative control. After incubation in a 5% CO_2_ humidified chamber at 37°C for 24 h, the wells were washed vigorously five times with PBS and incubated with a biotinylated anti-human IFN-c antibody and horseradish peroxidase-conjugated avidin. Spots were visualized and analyzed using KS ELISPOT (Carl Zeiss, Jena, Germany). In the present study, the cutoff point for ELISPOT was determined according to previous studies; positive (+) ELISPOT represented a more than twofold increase of survivin-2B80–88 peptide-specific CD8 T cell IFNc-positive spots compared with HIV peptide-specific CD8 T-cell spots, whereas negative (–) represented a less than twofold increase [13, 14].

### 2.10. Statistical Analysis

Continuous variables were compared using the Student's *t*-test. Given the small size, we confirmed all results with the Mann-Whitney *U* test. Categorized variables were compared using Fisher's exact probability test. Overall survival rates (OS) were evaluated by the Kaplan-Meier method, and differences between two groups were compared using the log-rank test and Cox proportional hazards regression models. A value of *P* < 0.05 was considered to indicate statistical significance. The calculations were performed using Statview 5.0 (SAS Institute, Cary, NC).

## 3. Results

### 3.1. Patient Profile

Twenty-one patients were enrolled in Study 2 (Table 1). They consisted of 15 men and 6 women, whose age range was 36–77 years. Three patients did not receive chemotherapy before vaccination because they were unfit for cisplatin-based chemotherapy due to impaired renal function.

### 3.2. Safety

Six patients (cases 5, 6, 16, 17, 19, and 20) discontinued halfway through the protocol because of disease progression. The remaining 15 patients received the complete regimen including four vaccinations. None of the treatment interruptions was due to adverse effects of the vaccination. Peptide vaccination was well tolerated in all 21 patients. As shown in Table 2, no hematologic, cardiovascular, hepatic, or renal toxicity was observed. No other severe adverse events were observed during or after vaccination. As minor side effects, 14 patients (cases 1–6, 8–10, 12, 13, 15, 17, and 18) developed grade 1 fever, possibly due to IFN alpha, and 3 patients (cases 4, 7, and 18) developed grade 1 local skin reactions with redness and induration at the injection sites. No other severe adverse events were observed during or after vaccination.

### 3.3. Immunological and Clinical Responses

**Figure 2:**
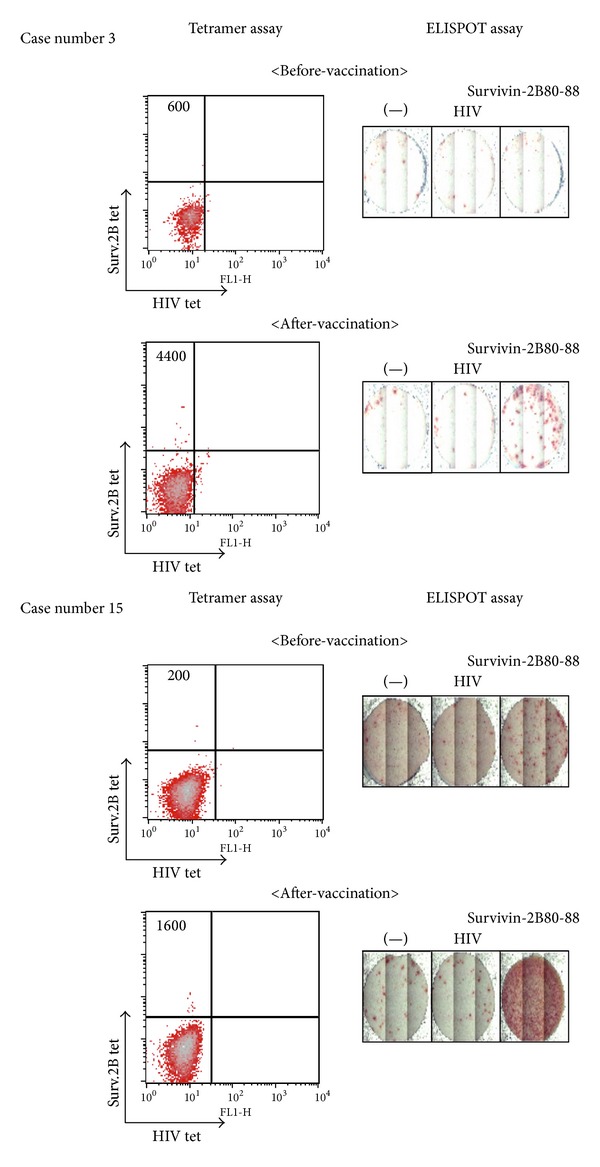
Representative illustration of immunological analysis in patients 3 and 15 who were treated with survivin-2B80–88 plus IFA with IFN alpha. Tetramer and ELISPOT analyses before and after vaccinations. The number in the tetramer analysis indicates survivin-2B80–88 peptide-specific CD8+ T cells among 10^4^ CD8+ T cells. ELISPOT: enzyme-linked immunosorbent spot; HIV: human immunodeficiency virus; HLA: human leukocyte antigen; IFA: incomplete Freund's adjuvant; IFN: interferon.

Representative illustrations of immunological analysis in cases 3 and 15 are shown in Figure 2, and Table 2 summarizes the immunological and clinical results. HLA-A24/survivin-2B80–88 peptide tetramer analysis revealed a significant increase in the peptide-specific CTL frequency of CD8-positive T cells after vaccination in 13 patients (cases 2, 3, 4, 6, 7, 9, 11, 12, 14, 15, 16, 19, and 21), as shown in Table 2. Of them, however, cases 6, 16, 19, and 21 were negative in the ELISPOT study. Thus, functional peptide-specific CTLs were induced in nine patients (42.8%) by this vaccination protocol. Radiographical examination revealed SD after four vaccinations in six patients (28.6%). All of them had an increase in the peptide-specific CTLs proven in both tetramer analysis and ELISPOT assay.

### 3.4. Impact of IFN Alpha in Combination with the Survivin-2B80–88 Peptide on Immunological Responses and Survival

**Figure 3:**
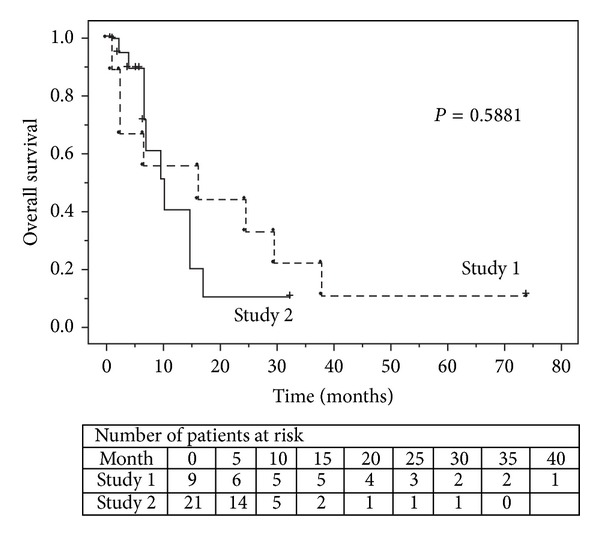
Kaplan-Meier estimated overall survival is shown for patients treated with survivin-2B80–88 peptide plus IFA (Study 1) versus survivin-2B80–88 peptide plus IFA in combination with IFA alpha (Study 2). IFA: incomplete Freund's adjuvant; IFN: interferon.

To assess the effect of additional IFN alpha, immunological and clinical outcomes were compared between Study 1 and Study 2. Baseline characteristics and immunological and clinical responses are shown in Table 3. There were no significant differences in either the induction of peptide-specific CTLs or radiographical responses. Furthermore, OS showed no significant difference between the two groups (Figure 3).

### 3.5. Impact of the Survivin-2B80–88 Peptide Vaccination with and without IFN Alpha on Survival

**Figure 4:**
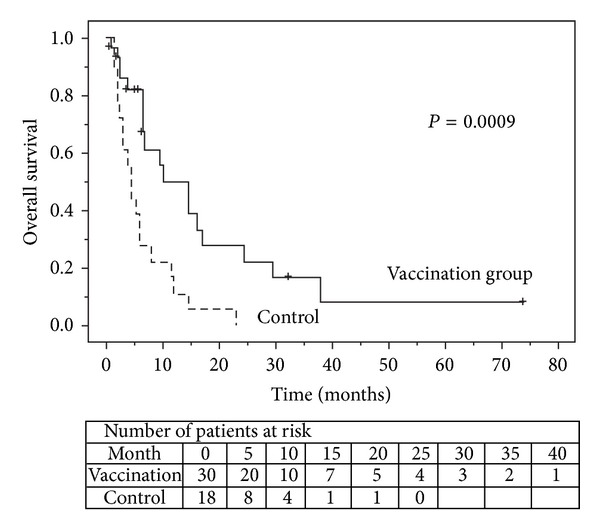
Kaplan-Meier estimated overall survival (OS) is shown for patients who received survivin-2B80–88 peptide vaccination with and without IFN alpha and did not receive survivin-2B80–88 peptide vaccination. A statistically significant difference in OS was identified between the two groups.

A total of 30 patients underwent the survivin-2B80–88 peptide vaccination in Study 1 and Study 2. During the course of these studies, 14 patients were excluded due to an ineligible HLA type and 4 patients eventually decided not to receive vaccination although eligible. These 18 patients were evaluated as a control group. Clinical characteristics were comparable between the vaccination group and control group, as shown in Table 4. The vaccination group had significantly better OS than the control group (*P* = 0.0009), as shown in Figure 4. Median survival times were 10.0 months and 4.5 months in the vaccination group and control group, respectively.  Table 5 lists the results of proportional hazards regression analysis used to test the predictive value of each variable for OS. In this multivariate model adjusted for age, ECOG PS, and the presence of visceral metastases, vaccination therapy was an independent predictive factor for better OS (*P* = 0.0088).

## 4. Discussion

Survivin-2B80–88 vaccination therapy is safe and confers induction of peptide-specific CTLs in patients with metastatic urothelial cancers according to the results of Study 1 [12]. In Study 2, we used a combination protocol of survivin peptide vaccination with IFN alpha in an attempt to enhance the immunogenicity, as with colorectal [13] and pancreatic cancers [14]. The protocol was safe and well tolerated with no severe adverse effects, as in the case of colorectal and pancreatic cancers [13, 14]. Fever was the most frequent adverse effect and may have resulted from the use of interferon alpha. However, we could not find any benefit of additional interferon alpha in urothelial cancer patients in terms of enhancing the immunogenicity of the survivin-2B80–88 peptide. In a previous study, peptide-specific CTLs were induced in 50% of patients with colorectal cancer by survivin-2B80–88 peptide vaccination in combination with interferon alpha, but in 0% by that without interferon alpha [13]. On the other hand, survivin peptide vaccination without interferon alpha induced peptide-specific CTLs in 67% of urothelial cancer patients [12]. IFN alpha may be an insufficient adjuvant for further enhancement of the immunogenicity of survivin-2B80–88, and improvement of the protocol is required. Besides IFN alpha, other cytokines such as interleukin (IL)-2, IL-4, IL-15, and granulocyte macrophage colony-stimulating factor are expected to have effects leading to stronger immune responses in both the induction and effector phases [16]. Furthermore, blockade of negative regulation of the immune response is considered to be an important strategy to enhance the immunological and clinical responses of peptide vaccination therapy [16, 17]. Low-dose cyclophosphamide [18, 19] and an anti-CD25 [20] antibody are employed to suppress regulatory T cells, and low-dose gemcitabine is promising to suppress myeloid-derived suppressor cells [21]. These agents might be effective to enhance the immunological response and clinical efficacy in survivin-2B-80-88 peptide vaccination therapy for urothelial cancer.

In cancer vaccination therapy, tumor shrinkage is not expected and may not be an appropriate endpoint for evaluation of the efficacy of cancer immunotherapy [22]. Although neither CR nor PR after the vaccination was observed in our series, all six patients with SD also had increases in CTLs. SD can be considered to be a result of immunological responses to the survivin-2B80–88 peptide vaccine. Therefore, the results of the current study suggest that survivin-2B80–88 peptide vaccination therapy potentially provides survival benefit for patients with metastatic urothelial cancer. However, this study had only a small number of subjects. Although the control group was comparable, patients were not randomized. To confirm the efficacy for survival, a larger randomized clinical trial is necessary.

There is no standard therapy for metastatic urothelial cancers refractory to standard chemotherapy [2, 3]. In addition, most second-line chemotherapy regimens under investigation have severe adverse events, which can impair patients' quality of life and are often associated with life-threatening adverse effects [4–6]. On the other hand, the results of the current study suggest that survivin-2B80–88 peptide vaccination therapy is safe and well tolerated and may potentially have clinical benefits in selected patients. Thus, survivin-2B80–88 peptide vaccination appears to be a promising treatment strategy for metastatic urothelial cancers refractory to standard chemotherapy.

## 5. Conclusions

Although survivin-2B80–88 peptide vaccination in combination with IFN alpha is safe and well tolerated, the effects of additional IFN alpha are unclear. According to the results of pooled data analysis of Study 1 and Study 2, survivin-2B80–88 peptide vaccination therapy potentially has clinical effects; thus, it may be a promising therapy for selected patients with metastatic urothelial cancers refractory to standard chemotherapy.

## Figures and Tables

**Table 1:** Profiles of patients with advanced urothelial cancer enrolled in Study 2.

No.	Age	Sex	Primary site	Recurrence site	ECOG PS	Prior chemotherapy (number of cycles)
1	72	f	UUT	LN in neck, mediastinum, and abdomen	1	MVAC (5), TIN (2)
2	36	m	Bladder	Abdominal LN Bone	0	MVAC (2), TIN (1)
3	61	m	UUT	Pelvic soft tissue	0	GC (3), TIN (2)
4	75	m	Bladder	Abdominal LN	0	MVAC (2)
5	76	m	UUT	Renal pelvis, urethra	2	GC (3), TIN (1)
6	60	f	UUT	Abdominal LN	1	MVAC (3)
7	72	f	UUT	Mediastinal LN	1	MEC (1), GEM (1), TIN (2)
8	77	m	Bladder	Lung	0	None
9	75	f	UUT	Pelvic soft tissue	1	None
10	68	m	UUT	Abdominal LN Lung	0	GC (4)
11	72	m	Bladder	Pelvic LN, liver	0	TG (1)
12	58	m	Bladder	LN in neck, abdomen, and pelvis	0	GC (2), TIN (2)
13	64	m	Bladder	LN in abdomen and pelvis	1	GC (2)
14	73	f	Bladder	Lung, liver, and bone	0	GC (3), TIN (2)
15	62	m	Bladder	Lung	1	GC (1), GCar (1)
16	74	m	Bladder	Abdominal LN	2	GC (4), TIN (2)
17	53	m	UUT	Lung, subcutaneous	2	None
18	61	m	Bladder	Lung	1	GC (6), TIN (3)
19	56	m	Bladder	Abdominal LN, liver	1	GCar (2)
20	63	f	Bladder	Abdominal LN, lung, and liver	1	GC (4)
21	73	m	Bladder	Abdominal LN, liver	0	GC (4)

UUT: upper urinary tract; LN: lymph node; ECOG PS: Eastern Cooperative Oncology Group performance status; MVAC: methotrexate, vinblastine, adriamycin, and cisplatin; TIN: paclitaxel, ifosphamide, and nedaplatin; GC: gemcitabine and cisplatin; MEC: methotrexate, etoposide, and cisplatin; GEM: gemcitabine; TG: paclitaxel and gemcitabine; GCar: gemcitabine and carboplatin.

**Table 2:** Summary of clinical and immunological responses to vaccination with survivin-2B80-88 peptide, IFA, and IFN alpha.

No.	Adverse events (Grade)*	Tetramer staining^†^	ELISPOT^‡^	Clinical response	Followup (months)	Outcome
		After/before vaccination	After/before vaccination			
1	Fever (1)	3100/3300	98/3	PD	6.5	DOD
2	Fever (1)	2700/600	31/20	SD	14.5	DOD
3	Fever (1)	4400/600	62/36	SD	17.0	DOD
4	Fever (1) Induration at injection site (1)	16400/1700	49/12	SD	32.5	AWD
5	Fever (1)	500/10900	29/8	PD	2.0	DOD
6	Fever (1)	2000/300	32/29	PD	4.0	DOD
7	Induration at injection site (1)	4100/0	41/15	PD	6.5	DOD
8	Fever (1)	2100/2000	49/33	PD	14.5	DOD
9	Fever (1)	2000/500	65/21	SD	7.0	DOD
10	Fever (1)	0/4500	53/6	PD	6.5	AWD
11	None	38900/0	10/0	PD	10.0	DOD
12	Fever (1)	2400/0	117/80	SD	6.0	AWD
13	Fever (1)	0/0	28/9	PD	9.5	DOD
14	None	1200/800	95/14	PD	4.0	AWD
15	Fever (1)	1600/200	616/68	SD	8.5	AWD
16	None	700/300	11/63	PD	5.5	DOD
17	Fever (1)	200/400	39/0	PD	5.5	AWD
18	Fever (1) Induration at injection site (1)	3700/4600	61/32	PD	4.0	AWD
19	None	1400/200	7/5	PD	1.0	AWD
20	None	900/800	25/0	PD	2.0	AWD
21	None	2000/300	0/0	PD	2.0	AWD

*Adverse events were recorded using the National Cancer Institute Common Terminology Criteria for Adverse Events version 4.0 (CTCAE v4.0). ^†^Cytotoxic T-lymphocyte (CTL) frequency before and after treatment in patients was assessed with an HLA-A24-restricted survivin-2B80-88 (AYACNTSTL) peptide tetramer. An HLA-A24-restricted HIV peptide (RYLRDQQLL) tetramer was used as a negative control. The number of survivin-2B80-88 peptide tetramer-positive but HIV peptide-negative CTLs in 10^4^ CD8 T cells is shown. ^‡^Interferon gamma secretion of pre- and postvaccinated patients' CD8 T cells was assessed with enzyme-linked immunosorbent spot (ELISPOT) assay using T2-A24 cells pulsed with survivin-2B80-88 peptide. The numbers of spots in 5 × 10^3^ CD8 T cells are shown. SD: stable disease; PD: progressive disease; DOD: dead of disease; AWD: alive with disease.

**Table 3:** Baseline characteristics and immunological responses in Study 1 and Study 2.

	Study 1	Study 2	*P* value
*n*	9	21	
Age	58.1 ± 9.1	65.7 ± 10.3	0.0593
Sex (male/female)	4/5	15/6	0.2252
Primary site (UUT/bladder)	1/8	11/10	0.0492
Visceral metastases	4 (44.4%)	11 (52.4%)	0.9999
Prior chemotherapy	9 (100%)	18 (85.7%)	0.2320
ECOG PS (0/1/2)	4/5/0	10/9/2	0.9999
Induction of CTLs	5 (71.4%)*	9 (42.8%)	0.3845
Non-PD in clinical response	2 (33.3%)^†^	6 (28.6%)	0.6424

*Immunological results were obtained in 7 of 9 cases. ^†^Clinical responses were assessed in 6 cases. UUT: upper urinary tract; ECOG PS: Eastern Cooperative Oncology Group performance status; CTL: cytotoxic T-lymphocyte; PD: progressive disease.

**Table 4:** Clinical characteristics of vaccination group and control group.

	Vaccination group	Control group	*P* value
*n*	30	18	
Age	63.5 ± 10.2	66.4 ± 10.3	0.3355
Sex (male/female)	19/11	15/3	0.1956
Visceral metastases	15 (50.0%)	12 (66.7%)	0.3693
Number of visceral metastatic sites	0.63 ± 0.76	1.00 ± 0.84	0.1417
Prior chemotherapy	27 (90.0%)	17 (94.4%)	0.9999
ECOG PS			
0	13 (43.3%)	8 (44.4%)	0.4002
1	14 (46.7%)	6 (33.3%)	
2	3 (10.0%)	4 (22.3%)	

ECOG PS: Eastern Cooperative Oncology Group performance status.

**Table 5:** Multivariate proportional hazards regression model for overall survival.

Variable	HR	95% CI	*P* value
Age: <65 versus ≥65 years	0.782	0.343–1.784	0.5591
ECOG PS: 0 versus ≥1	0.335	0.160–0.703	0.0038
Visceral metastases: no versus yes	0.599	0.284–1.263	0.1782
Vaccination: yes versus no	0.308	0.127–0.743	0.0088

ECOG PS: Eastern Cooperative Oncology Group performance status; HR: hazard ratio; CI: confidence interval.
